# Stretta Radiofrequency Treatment for GERD: A Safe and Effective Modality

**DOI:** 10.1155/2013/783815

**Published:** 2013-09-02

**Authors:** Mark Franciosa, George Triadafilopoulos, Hiroshi Mashimo

**Affiliations:** ^1^Center for Swallowing and Motility Disorders, VA Boston Healthcare System, Harvard Medical School, Boston, MA 02132, USA; ^2^Division of Gastroenterology and Hepatology, Stanford University School of Medicine, Stanford, CA 94040, USA

## Abstract

Gastroesophageal reflux disease is one of the leading gastrointestinal disorders. Current treatments include lifestyle modifications, pharmacological therapies, surgical fundoplications, and, more recently, endoscopic procedures. The rising concern of long-term side effects of the popular proton-pump inhibitors and the more recent evidence raising doubts about the durability of fundoplication have spurred reinterest in endoscopic procedures to treat reflux disorders. In the aftermath of several innovative antireflux procedures that were introduced and failed clinically or financially over the past decade, there is lingering confusion regarding the merits of the presently available interventions. This paper focuses on one endoscopic procedure, Stretta, which now enjoys the longest experience, a recent meta-analysis, and robust data supporting its safety, efficacy, and durability. Stretta reduces esophageal acid exposure, decreases the frequency of transient lower esophageal relaxation, increases patient satisfaction, decreases medication use, and improves quality of life. As such, this procedure remains a valuable nonsurgical treatment option in the management of gastroesophageal reflux disease.

## 1. The Burden of Gastroesophageal Reflux Disease

Gastroesophageal reflux disease (GERD) is the most common digestive disorder affecting one third of the population worldwide and resulting in 4 to 5 million physician visits annually. It results primarily from the loss of an effective antireflux barrier against the retrograde movement of gastric contents into the distal esophagus. The average incremental cost in the United States to an employer for an employee with GERD in 2007 was estimated to be $ 3,355 per year including medical costs, prescription drug costs, and indirect costs such as absenteeism and disability [1]. Furthermore, a significant financial burden on medical care comes from hospital admissions due to acid-induced noncardiac chest pain. Uncontrolled GERD results in a significant reduction in quality and productivity at work. GERD is also a risk factor for esophageal adenocarcinoma that is becoming increasingly prevalent and has the fastest rising incidence of any cancer [2]. The current treatment for GERD consists of lifestyle modifications, pharmacological therapies, endoscopic procedures, and surgical interventions. The initial management of GERD includes lifestyle modifications, such as elevating the head of the bed, dietary modifications, restricting alcohol, and managing obesity. Pharmacological management typically consists of the use of H2 blockers and, in most cases, proton-pump inhibitors (PPIs). Although medical therapy with PPIs is effective in most patients, there are increasing concerns regarding the long-term use of these drugs. These include interaction with a number of cardiac medications such as clopidogrel [3], association with osteoporotic fractures [4], hospital-acquired diarrhea and pneumonia, hypomagnesemia, and vitamin B12 malabsorption [5]. In addition, prolonged PPIs use has been associated with chronic atrophic gastritis in patients infected with *H. pylori* [6]. In the recent years, a significant number of patients with GERD are found to be refractory to PPIs therapy despite even twice daily use of these drugs [7]. Surgical options for GERD also have their limitations including increased costs, hospitalization, up to 10% complication rate, and 28-day recovery [8]. Furthermore, the durability and side effects of fundoplication have fallen short of expectations. Recent 5-year data from the LOTUS trial suggests that 15%–20% of those who have undergone fundoplication may have GERD symptoms [9]. 

## 2. Advent of Nonsurgical Antireflux Devices

 Since the early 2000's, several devices have been developed for the endoscopic treatment of GERD, using approaches such as sewing, transmural fasteners, endoscopic staplers, and thermal treatment using radiofrequency energy. Other devices involving injection, Enteryx (Boston Scientific, Boston, MA, USA) or implantation of foreign materials, Gatekeeper reflux repair system (Medtronic, Inc., Minneapolis, MN, USA) at the esophageal junction are no longer used. Devices that are currently commercially available for the endoscopic treatment of GERD in the United States include the following: EndoCinch (C. R. Bard, Inc., Murray Hill, NJ, USA); EsophyX (EndoGastric Solutions, Redwood City, CA, USA); Stretta (Mederi Therapeutics, Greenwich, CT, USA); and SRS Endoscope (Medigus, Omer, Israel). These are summarized in Table 1. Of these, Stretta, which applies radiofrequency energy to the lower esophageal sphincter (LES), has the longest experience in the treatment of GERD. 

## 3. What Is Stretta?

**Figure 1:**
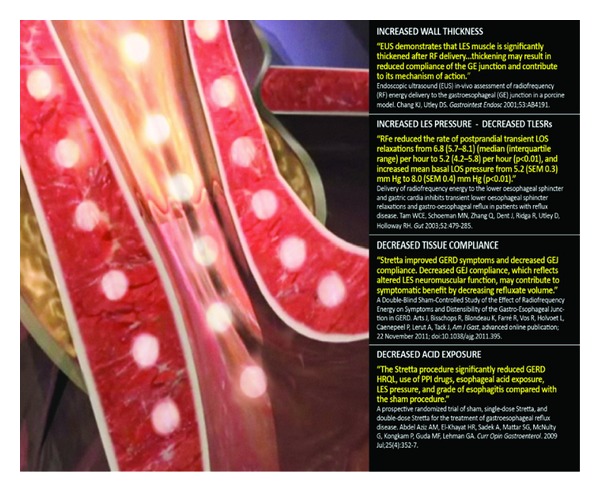
Stretta radiofrequency treatment mechanism of action (with the permission of Mederi Therapeutics, copyright 2013).

The Stretta procedure involves the application of controlled radiofrequency (RF) energy to the LES region. The procedure, approved by the Food and Drug Administration in the United States in 2000, uses a flexible catheter with a balloon-basket assembly and nickel-titanium needle electrodes to deliver the radiofrequency energy into the esophageal wall and LES complex, while irrigating the overlying mucosa to prevent heat injury.  Figure 1 illustrates the established mechanisms of action of Stretta. 

Initial animal studies used porcine and canine models and showed a thickening of the LES, decreased transient lower esophageal relaxations (TLESRs), and decreased reflux events [10]. Multiple studies have demonstrated the safety and efficacy of the Stretta procedure for GERD therapy. Some studies had mixed results of its effectiveness and durability [11]. Despite four randomized clinical trials, more than 60 prospective trials and more than 800 patients followed post-Stretta procedure for 12 to 48 months, and there remain unanswered questions, overstated myths, and underappreciated realities about options in management of GERD. Such questions include whether PPIs are truly effective and safe, whether Stretta causes a stricture or neurolysis of the LES, whether Stretta effectively decreases acid exposure and improves symptoms and quality of life, and whether the improvements are durable over time. In this paper we address these questions and conclude that Stretta is a safe and effective alternative to medical management or surgical management in selected patients. 

## 4. Myths about Stretta

### 4.1. Myth: Proton-Pump Inhibitors Effectively Control Symptoms in All Patients with GERD

PPIs comprise a class of drugs widely used for the treatment of GERD. Their mechanism of action involves inhibition of the H-K ATPase enzyme that is present in gastric mucosal parietal cells. This enzyme is responsible for the secretion of hydrogen ions in exchange for potassium in the gastric lumen, and its inhibition decreases gastric acidity. First introduced in the late 1980's, PPIs were the most potent inhibitors of gastric acid secretion available, with efficacy superior to histamine-2 receptor antagonists. Because they effectively alleviate gastric-peptic symptoms and facilitate healing of inflamed or ulcerated mucosa, current guidelines recommend their use for the treatment of GERD. PPIs are also well tolerated, with side effects occurring at a rate of 1%–3% and with no significant differences among the various agents. Such side effects most commonly include headaches, nausea, abdominal pain, constipation, flatulence, diarrhea, rash, and dizziness. However, over the past decade, an increasing number of studies has shown that GERD symptom control is not as optimal as originally thought and marketed. A post hoc analysis of 5,794 patients from four randomized double-blind studies revealed that partial heartburn relief was experienced with the use of PPIs in 19.9% of patients with nonerosive reflux disease and in 14% of patients with reflux esophagitis [7]. Another study reported that only 61% of patients on PPIs with nonerosive esophageal reflux disease experienced resolution of heartburn [12]. 

### 4.2. Myth: PPIs Use Is Safe

Over the past decade, several potential adverse effects of long-term PPIs use had generated great concerns: B12 deficiency; iron deficiency; hypomagnesemia; increased susceptibility to pneumonia; enteric infections; fractures; hypergastrinemia; and drug-drug interactions [4]. This has led many patients with GERD either to self-discontinue therapy resulting in symptomatic recurrence or to solicit alternative methods to control their symptoms. Miyamoto and colleagues followed a cohort of 44 patients over 5 years and found that only 77% had improvement in their reflux symptoms [13]. Lundell and colleagues followed a cohort of 53 patients randomized to PPIs versus fundoplication; only 45% had continuous remission up to 12 years after randomization to the PPIs arm [14]. 

### 4.3. Myth: Fundoplication Effectively Controls Reflux Symptoms

Fundoplication as a means of controlling GERD symptoms over a sustained period of time has shown poor results. Lundell and colleagues followed a cohort of 144 patients for 7 years after fundoplication examining for recurrence of GERD symptoms and the need to resume medical management of reflux symptoms. They found that 34% had symptomatic relapse, and many of them required medical management [15]. Smith and colleagues followed a cohort of 1892 patients for 10 years post fundoplication and found that 17% had resumed using antisecretory medications [16]. Spechler and colleagues followed a cohort of 38 patients for 10 years after fundoplication and found that 62% were using antisecretory medications [17]. Oelschlager and colleagues followed a cohort of 289 patients for 5 years and found that 61% of them were taking some forms of antacid [18]. 

### 4.4. Myth: Stretta Causes Distal Esophageal Strictures

Although the exact mechanism of action of Stretta in relieving symptoms of acid reflux is unknown, one potential mechanism is that it decreases the number of TLESRs [19]. The latest theory suggests that this is accomplished by a structural rearrangement of the smooth muscle and redistribution of the interstitial cells of Cajal in the smooth muscle of the LES [20]. Stretta was designed to minimize damage to the esophagus. The four-channel radiofrequency (RF) generator and catheter system delivers pure sine-wave energy (465 kHz, 2 to 5 watts per channel, and 80 volts maximum at 100 to 800 ohms). Each needle tip incorporates a thermocouple that automatically modulates power output to maintain a desired target (muscle) tissue temperature. Maintaining lesion temperatures below 50°C minimizes the collateral tissue damage due to vaporization and high impedance values. Temperature is similarly monitored with a thermocouple at each needle base, and power delivery ceases if the mucosal temperature exceeds 50°C or if impedance exceeds 1000 mOhms [19]. Maintaining tight temperature control prevents mucosal damage to the distal esophagus and gastroesophageal junction thus preventing stricture formation. A recent double-blind sham-controlled study of 22 patients showed that administration of sildenafil, an esophageal smooth muscle relaxant, normalized the gastroesophageal junction compliance to pre-Stretta levels, arguing against GE junction fibrosis as an underlying mechanism [19]. 

### 4.5. Myth: Stretta Causes Neurolysis in the Distal Esophagus

DiBaise and colleagues followed a cohort of 18 patients 6 months after Stretta and found no adverse effects on abdominal vagal function and no significant changes in any esophageal motility parameter; however, a trend was noted toward a reduction in the number of TLESRs induced by gastric air distension (3.5/h versus 1.0/h; *P* = 0.13). No detrimental effects on peristalsis or swallow-induced LES relaxation pressure were seen [21]. Arts and colleagues also followed a cohort of 13 patients for 6 months after Stretta and found that esophageal peristalsis (low-amplitude peristalsis in the same three patients), resting LES pressure (18.2 ± 2.0 mm Hg; NS), and swallow-induced relaxations were not significantly altered by the radiofrequency energy delivery procedure, which also argues against the theory of neurolysis [22]. 

### 4.6. Myth: Stretta Does Not Decrease Esophageal Acid Exposure

**Figure 2:**
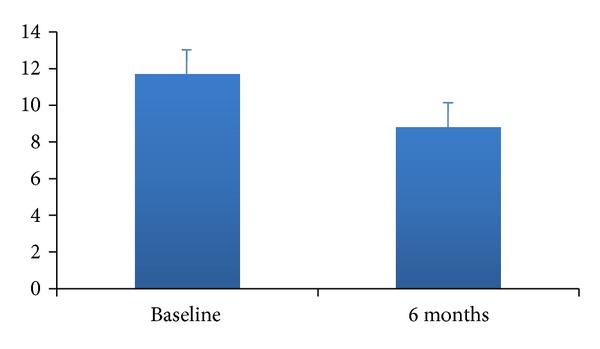
Acid exposure (% pH < 4.0) before and after Stretta.

Several studies have shown a decrease in esophageal acid exposure after Stretta. Arts and colleagues followed a cohort of 13 patients over 6 months, and all patients underwent repeat pH monitoring 6 months after the procedure. One measurement was technically inadequate and not interpretable. In the evaluable patients, esophageal pH monitoring was significantly improved, from 11.6% ± 1.6% to 8.5% ± 1.8% of the time at pH < 4 (*P* < 0.05) (Figure 2). Normalization of the pH monitoring (<4% of the time at pH < 4) occurred in only three patients. The DeMeester score showed a similar improvement, from 46.8 ± 7.3 to 35.6 ± 6.7 (*P* = 0.01) [21]. Aziz and colleagues showed similar results from their prospective randomized sham study of 36 patients, which showed significant reduction in esophageal acid exposure [23]. Not all studies have come to the same conclusion. DiBaise and colleagues followed a cohort of 18 patients after Stretta for 6 months and found that there were no adverse effects on vagal function and esophageal motility. There were an improvement in symptoms, a decreased antacid use, and decreased TLESRs, but no significant difference in esophageal acid exposure [21]. Even though decrease in acid exposure was not achieved in this study, Stretta did accomplish the primary goals of GERD treatment which are to improve symptoms, improve quality of life, and decrease medication use. Although this study did not show decreased acid exposure, there are multiple other studies that did show a decrease, and it is important to look at the entire body of research showing, in many cases, improvement in acid exposure. 

The recently published meta-analysis by Perry and colleagues evaluated 18 studies and 1441 patients and showed a significant reduction in esophageal acid exposure after Stretta. Preprocedure and postprocedure esophageal pH studies were documented in 11 of the 20 studies. The DeMeester score improved from 44.37 ± 93 before Stretta to 28.53 ± 33.4 after Stretta over an average period of 13.1 months in 267 patients across 7 studies (*P* = 0.0074). The esophageal acid exposure was reported in 11 studies comprising of 364 patients over a mean follow-up period of 11.9 months. Esophageal acid exposure decreased from a mean of 10.29% ± 17.8% to 6.51% ± 12.5% (*P* = 0.0003) [11]. 

### 4.7. Myth: Stretta Relieves GERD Symptoms by Placebo Effect 

Due to the lack of certainty around the mechanism by which Stretta relieves GERD symptoms, there is a misconception that Stretta works by placebo effect. Arts and colleagues performed a double-blind sham-controlled study showing that Stretta decreases LES compliance, which likely mitigates inappropriate LES relaxations, the most common underlying cause of GERD. This study also showed that there was a significant improvement in symptoms as compared with the patients who received the sham procedure [19]. Further evidences that Stretta does not work by placebo effect are the studies showing decreased esophageal acid exposure after Stretta [22, 23]; see Table 2 for the summary of the myths about Stretta. 

## 5. Realities of Stretta

### 5.1. Reality: Stretta Improves Quality of Life and Patient Satisfaction

**Figure 3:**
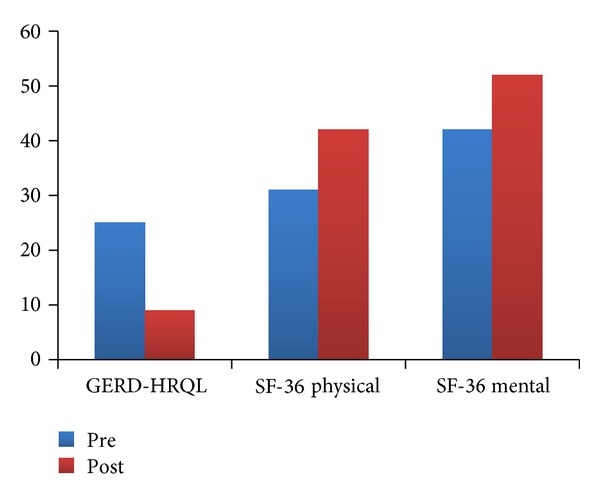
Scores before and after Stretta.

There have been numerous studies showing that patients treated with Stretta have a significant improvement in quality of life. In the meta-analysis by Perry, 18 studies containing 433 patients evaluated the effect of treatment on patient quality of life (QoL) using the GERD-HRQL scale with an average follow-up interval of 19.8 months. The QoL scores improved from 26.11 ± 27.2 at baseline to 9.25 ± 23.7 after treatment (*P* = 0.0001). QoL scores were collected from 4 studies comprising 250 patients and were improved from 3.3 ± 5.9 to 4.97 ± 4.9 at a mean follow-up interval of 25.2 months (*P* = 0.001). SF-36 was utilized to assess global QoL of the patient population in 6 studies. A total of 299 patients responded to the SF-36 physical form, during a mean follow-up period of 9.5 months, demonstrating an improvement from 36.45 ± 51.6 at baseline to 46.12 ± 61.9 after procedure (*P* = 0.0001). Two hundred sixty-four patients in 5 of the 6 studies responded to the SF-36 mental form demonstrating improvement from 46.79 ± 20.5 to 55.16 ± 17.6 at 10-month followup (*P* = 0.0015) [11] (Figure 3).

### 5.2. Reality: Stretta Decreases Acid Reflux Symptoms and Medication Use

**Figure 4:**
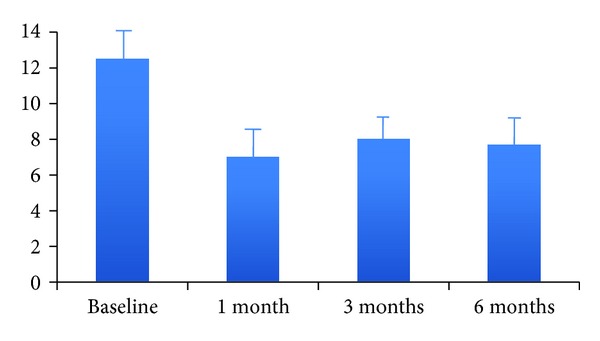
Reflux symptoms 6 months after Stretta.

**Figure 5:**
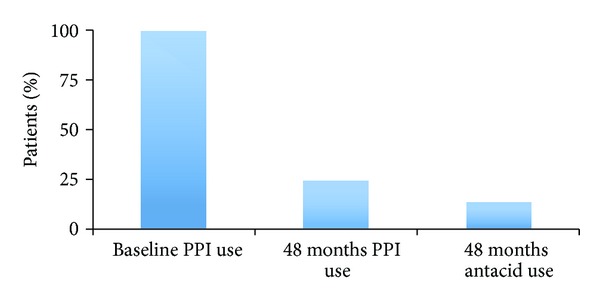
PPI and antacid use 48 months after Stretta.

There have been several studies showing a significant decrease in medication use after Stretta. Triadafilopoulos and colleagues conducted a nonrandomized, prospective, and multicenter study that included 118 patients treated with Stretta for GERD. Follow-up information was available for 94 patients (80%) at 12 months; the proportion of patients requiring PPIs fell from 88% to 30%. There was also an improvement in quality of life scores and reduction in esophageal acid exposure [20]. In another trial by Liu and colleagues of 90 patients with nonerosive or mildly erosive disease, the onset of GERD symptom relief after Stretta was less than two months in 70.0% and two-to-six months in 16.7%, while there was a significant improvement in GERD symptoms and patient satisfaction (Figure 4). Medication usage decreased significantly from 100% of patients on PPIs therapy at baseline to 76.7% of patients showing elimination of medication use or only as-needed use of antacids/H2-receptor antagonists at 12 months [24]. Dughera and colleagues reported similar results in 48-month follow-up data for 56 out of 69 patients who were treated with Stretta. RF treatment significantly improved heartburn scores, GERD-related quality of life scores, and general quality of life scores at 24 and 48 months in 52 out of 56 patients (92.8%). At 48 months, 41 out of 56 patients (72.3%) were completely off PPIs (Figure 5). Morbidity was minimal, except for one patient who developed transient gastroparesis [25]. 

### 5.3. Reality: Stretta Is Safe

The recently published meta-analysis by Perry revealed that the most common complications encountered after the Stretta procedure were gastroparesis and erosive esophagitis. These are known to be transient and reversible. Early reports of esophageal perforations were attributed to operators' inexperience, and no such grave complications have been reported since then [10]. In a study of 77 patients who had the Stretta procedure, none had esophageal perforation, dysphasia, or severe gas bloating or stricture, documenting low complication rates for mild fever (2/24:8%), pneumonia (1/24:4%), transient dysphasia (3/24:12.5%), abdominal pain (2/24:8%) and 0% mortality [26]. Complication rates compare favorably with those of surgical interventions that appear to be around 4%, for laparoscopic procedures and 9% for open fundoplications [27]. There have been only 29 adverse events for more than 15,000 preformed procedures reported to the FDA with the last being in 2005. There have been no adverse events reported since the latest upgrades of the Stretta device in 2005. The upgrades include more sensitive temperature controls, easier user interface, and newer ablation prongs. 

### 5.4. Reality: Stretta Is Durable

**Figure 6:**
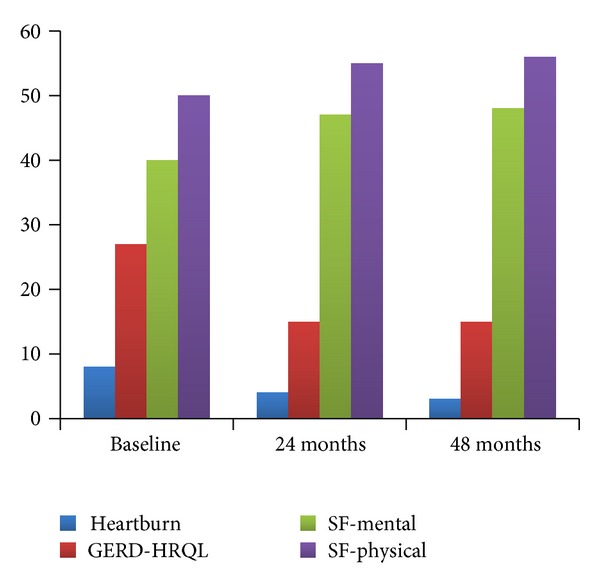
Symptoms and quality of life after Stretta.

There have been several long-term studies examining the durability of Stretta. One of the longest follow-up studies has been that by Noar and colleagues who showed that, in 109 patients with 48 months of followup, 75% of patients showed statistically significant reduction in PPIs usage, and there were significant improvements in patient satisfaction and heartburn scores [28]. Another study by Reymunde and colleagues followed a cohort of 83 patients for 48 months and found statistically significant improvement in GERD symptom scores and GERD-QoL scores, besides reporting that daily medication use was needed by only 13.6% of patients at 48 months, compared with 100% prior to treatment [29] (Figure 6). Recently, Dughera reported on 56 patients who also reached 48 months of followup and had significantly improved heartburn scores, GERD-specific QoL scores, and general QoL scores at 24 and 48 months in 52 (93%) of patients. At 48 months, 41 patients (72%) were completely off PPIs [25]. At 8 years, 60% of available patients were still not using PPIs [30]. This compares favorably with outcomes after fundoplication, showing that nearly 60% undergoing surgery were back on PPIs after 8 years. 

### 5.5. Reality: Stretta Improves Gastric Emptying

Growing clinical evidence shows that delayed gastric emptying (gastroparesis) may be a factor associated with severe reflux, dyspepsia, or both. Gastroparesis, concomitant in 25% of patients with gastroesophageal reflux disease (GERD), has been shown to improve after Stretta. Radiofrequency treatment for GERD may potentially correct GERD-associated gastroparesis and resultant reflux failures despite the twice daily use of PPIs. Noar and colleagues showed that at 6 months after Stretta procedure gastric emptying scores had improved significantly, with the percentage of solid food emptied at 90 min improving from 41% to 66% (*P* < 0.0001) and at 120 min improving from 55% to 84%. Significant improvements were seen at all time intervals. Overall, 23 patients (74%) experienced normalization of gastric emptying, and 4 patients improved but remained abnormal. Four patients showed no improvement on their gastric emptying scans, with one patient electing to undergo a Nissen procedure. All of the patients had a 1-year symptom follow-up assessment, which showed significant improvements in GERD-related quality of life, dyspepsia, and heartburn scores [31]. 

### 5.6. Reality: Stretta Has Limitations

One of the limitations of Stretta is that it has not proven to be cost effective. In a study by Comay et al., which followed a cohort of patients for 5 years after being randomized to either once daily PPIs therapy, fundoplication, or Stretta, this cohort was evaluated for quality-adjusted life years, symptom-free months, and cost effectiveness. Their results showed that the PPIs procedure was the most cost effective strategy depending on the price of omeprazole per pill. If the price of omeprazole was over $2.00 per pill, then Stretta was deemed the most cost-effective of the three strategies. The costs in this study were reported in Canadian dollars and based on costs in the Canadian health system. The estimated cost in this study of 5 years of PPIs use was $2394.10, the cost of Stretta was $3,239.30, and the cost of fundoplication was $7394.70 [32]. There is great variability in the cost of PPIs in the United States. At the time of this publication, the average retail price per pill in one major pharmacy chain was for $2.63 per pill omeprazole, for $4 per pill pantoprazole, and for $8.30 per pill esomeprazole. The hidden cost the patient must also take into consideration is the increasing number of side effects of PPIs that are being reported and the increasing appreciation of treatment failures [12]. Although Stretta has been associated with 29 complications in over 15,000 cases, including 5 esophageal perforations early in its launch, no serious adverse events have been experienced since the modified generator and catheter in 2011 under Mederi Therapeutics were used. 

 Gastroparesis is a side effect of Stretta. Dughera and colleagues found that only 1 out of 56 patients treated with Stretta developed gastroparesis, and this resolved in 8 weeks [25]. Noar and colleagues showed that Stretta improved gastroparesis in a study where they followed a cohort of 31 patients with gastroparesis 6 months after Stretta and found that 74% of patients have normalization of gastric emptying [31]. There have been more frequent cases of postsurgical gastroparesis that develops after surgical fundoplication for GERD. It is estimated that 4% to 40% of patients who undergo laparoscopic fundoplication develop intraoperative vagal damage to some degree [33].

### 5.7. Reality: Stretta Is Not for Every Patient with GERD

Stretta is ideal for patients with heartburn or regurgitation, patients who have adequate esophageal peristalsis, who have unsatisfactory GERD control with PPIs therapy, patients who have 24-hour pH monitoring demonstrating pathologic acid reflux, and patients who have nonerosive reflux disease or grade A or B esophagitis. The patients who are not considered good candidates for the Stretta procedure include those patients with a greater than 2 cm long hiatal hernia, patients who have significant dysphagia, patients who have grade C or D esophagitis, and patients who have inadequate esophageal peristalsis and incomplete LES relaxation with swallowing [34]. Thus, careful patient selection is important to assure benefit from this as well as other comparable procedures; see Table 2 for the summary of the realities of Stretta.

## 6. Conclusions

 In this paper, several randomized and prospective long-term studies have been presented that address concerns about the safety, tolerability, efficacy, and durability of Stretta that may make Stretta a more desirable treatment option than chronic PPI use or fundoplication in selected patients. 

## Figures and Tables

**Table 1:** Overview of treatments for GERD.

Procedures	Anesthesia	Cost	Number of cases worldwide	Years of experience	Number of centers using the device	FDA-reported adverse events
Stretta	Conscious sedation	$2000–3,500 per case	15,000	13	125	29
EsophyX	General gnesthesia	$7,000 per case	11,000	7	200	2
Medigus	General anesthesia	$3,200 per case	>100	2	2	0
Linx	General anesthesia	$12,000 per case	1000	5	70	0

**Table 2:** Summary of myths and realities concerning GERD treatment.

Myths	Realities
Proton-pump inhibitors effectively control symptoms in all patients with GERD.	Stretta improves quality of life and patient satisfaction.
Proton-pump inhibitor use is safe.	Stretta decreases acid reflux symptoms and medication use.
Fundoplication effectively controls reflux symptoms.	Stretta is not for every patient with GERD.
Stretta causes distal esophageal strictures.	Stretta is safe.
Stretta causes neurolysis in the distal esophagus.	Stretta is durable.
Stretta does not decrease esophageal acid exposure.	Stretta improves gastric emptying.
Stretta works by placebo effect.	Stretta has limitations.
